# Dihydromyricetin ameliorate postmenopausal osteoporosis in ovariectomized mice: Integrative microbiomic and metabolomic analysis

**DOI:** 10.3389/fphar.2024.1452921

**Published:** 2024-10-02

**Authors:** Lei Xu, Xianze Sun, Xiaoqiang Han, Hui Li, Xiaoqiong Li, Liying Zhu, Xin Wang, Jinjun Li, Haibiao Sun

**Affiliations:** ^1^ Department of Orthopedics, The First Hospital of Shanxi Medical University, Taiyuan, China; ^2^ State Key Laboratory for Managing Biotic and Chemical Threats to the Quality and Safety of Agro-Products, Zhejiang Academy of Agricultural Sciences, Hangzhou, China; ^3^ School of Life Sciences, University of Technology Sydney, Ultimo, NSW, Australia

**Keywords:** bone loss, dihydromyricetin, gut microbiota, ovariectomized mice, osteoprotegerin

## Abstract

The gut microbiota may help mitigate bone loss linked to postmenopausal osteoporosis by affecting the immune and inflammatory responses and the gut-bone axis. Dihydromyricetin (DMY), a natural flavonoid, has some anti-inflammatory and antioxidant properties. This study aimed to investigate the mechanisms underlying the amelioration of bone loss in ovariectomized (OVX) mice treated with various doses of DMY. Eight-week-old C57/BL6 mice underwent ovariectomy and received varying DMY doses over 8 weeks. Thereafter, femoral bone microarchitecture, serum biomarker levels, and colon samples were analyzed to assess bone metabolism and inflammatory and hormonal responses. Fecal samples were subjected to 16S rDNA sequencing, and short-chain fatty acids were quantified. An untargeted metabolomics approach was applied to both serum and fecal samples to investigate alterations in the intestinal microbiota and metabolic profiles following DMY treatment in the OVX mice. The results show high-dose DMY has anti-osteoporotic effects. Compared to the OVX group, the DMY-treated group showed enhanced bone mineral density and reduced inflammation and colonic damage levels. The DMY treatment altered the gut microbiota composition, including the relative abundances at both the phylum and genus levels. In addition, DMY treatment increased the production of acetate and propionate. Metabolomic analysis revealed differential regulation of 37 and 70 metabolites in the serum and feces samples, respectively, in the DMY-treated group compared to those in the OVX group, affecting the serotonergic signaling, arachidonic acid metabolism, and unsaturated fatty acid biosynthesis pathways. In conclusion, these findings indicate that DMY can ameliorate bone loss in OVX mice via the gut-bone axis.

## Graphical Abstract

**Figure:**
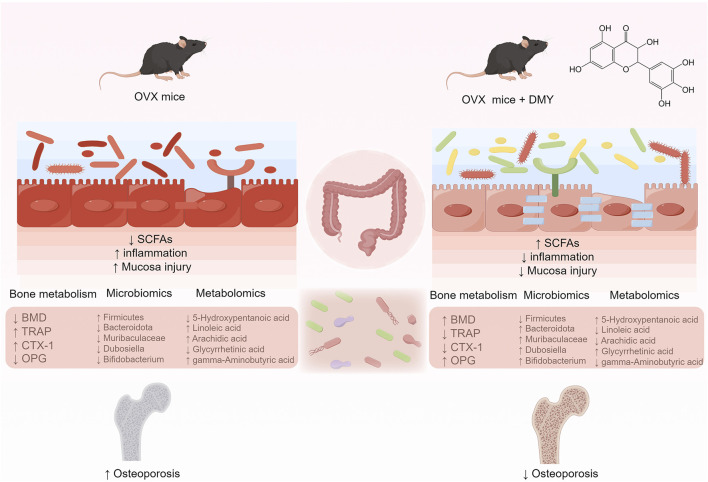
DMY can ameliorate bone loss in OVX mice via the gut-bone axis.

## 1 Introduction

Osteoporosis is a chronic bone metabolism-related disorder, associated with reduced bone mass, compromised bone architecture, and elevated risk of fractures, resulting in substantial morbidity and reduced productivity (Sarafrazi et al., 2021). Postmenopausal osteoporosis affects females and is precipitated by decreased estrogen levels, which enhance osteoclast activity and hasten bone resorption, which result in osteoporosis symptoms (Tella and Gallagher, 2014). Treatments for this condition currently include bisphosphonates, selective estrogen receptor modulators, and estrogen replacement therapy (Cappola and Shoback, 2016). However, these modalities may have adverse effects such as gastrointestinal discomfort, increased cardiovascular disease risk, and some oncogenic risks (Tella and Gallagher, 2014). Thus, novel, safer treatments are required.

Recent studies have suggested the role of the gut microbiota in human health, linking its dysregulation to conditions such as inflammatory bowel disease, obesity, diabetes, and cardiovascular diseases (Fan and Pedersen, 2021). The gut microbiota can modulate host immune responses, metabolic pathways, and inflammatory states, making it a potential therapeutic target for conditions including osteoporosis (Xu et al., 2022). The gut-bone axis describes the interplay between the gut microbiota and bone metabolism. The gut microbiota may affect bone density by modulating immune and inflammatory responses, and the gut-bone axis signaling (Guan et al., 2023). In addition, the gut microbiota produces metabolites such as short-chain fatty acids, which are crucial for maintaining intestinal barrier integrity and bone metabolism, affecting human health (Li et al., 2021). This evidence suggests that interventions at the level of the gut microbiota and associated metabolites may help improve osteoporosis outcomes.

Natural flavonoids may support disease prevention and treatment, given their anti-inflammatory, antioxidant, and immune-regulatory properties (Pei et al., 2020). In fact, flavonoids have demonstrated beneficial effects on bone health by modulating the gut microbiota balance (Wang et al., 2022). Natural flavonoids have a satisfactory safety profile, and their use may cause few side effects. Dihydromyricetin (DMY), sourced from *Ampelopsis grossedentata*, may affect the gut microbiota and intestinal metabolic processes, thus showing potential therapeutic effects in multiple diseases (Liu et al., 2019; Dong et al., 2021; Fan et al., 2018; Miao et al., 2023). Intraperitoneally administered DMY can inhibit osteoclast differentiation *in vitro* and ameliorate osteoporosis symptoms in ovariectomized (OVX) mice (Zhao et al., 2017). However, the dose-response effects of DMY on bone density and microarchitecture, and gut microbiota composition and metabolites produced, remain unknown.

This study aimed to use 16S rDNA gene sequencing and untargeted metabolomic analysis to investigate the impact of DMY on postmenopausal osteoporosis. This study used DMY at different doses and proposed the mechanisms of DMY effects on bone metabolism, which may include the regulation of the gut microbiota, augmentation of short-chain fatty acid (SCFAs) production, modulation of estrogen levels, and alteration of metabolic profiles. This study may advance the understanding of the gut-bone axis and suggest novel avenues for the management of postmenopausal osteoporosis.

## 2 Materials and methods

### 2.1 Animal models and therapeutic interventions

This study involved 50 female C57/BL6 mice, aged 8 weeks, supplied by Gempharmatech Co., Ltd. (Jiangsu, China). The mice were maintained in a controlled environment at a stable temperature (23°C–25°C) with a consistent 12-h light/dark cycle. The mice were randomly allocated to five groups, each containing 10 mice. The sham group (SHAM) underwent skin incisions, followed by suturing. The remaining 40 mice were ovariectomized (OVX). Anesthesia was induced by the intraperitoneal administration of sodium pentobarbital. After depilation and disinfection, a dorsal midline skin incision, approximately 0.5–1 cm in length, was made, followed by blunt separation of the skin and muscle layers to excise the ovaries.

The OVX mice were categorized into four groups: the model group (OVX) received daily 0.9% saline gavage; low-dose DMY group (DMY_L) received daily DMY gavage at 200 mg/kg; high-dose DMY group (DMY_H) received 400 mg/kg DMY, and treatment group (TREAT) received 1 mg/kg alendronate sodium daily. DMY and alendronate sodium were supplied by Shanghai Yuanye Bio-Technology Co., Ltd. (Shanghai, China). The duration of the experiment was 8 weeks. After the experiment, fecal samples were collected, and the anesthetized mice were euthanized with an intraperitoneal injection of pentobarbital sodium at a dose of 100 mg/kg. The anesthetized mice were placed in a supine position and secured on a wax board. An abdominal incision was made to immediately expose the abdominal cavity. The abdominal aorta was identified along the midline of the spine, positioned to the left of the inferior vena cava, with the left renal vein crossing its ventral surface. Using a syringe, blood was drawn directly from the abdominal aorta, with approximately 0.8–1 mL of blood collected per mouse for analysis. Following blood collection, femur and colon tissue samples were also collected. The study protocol was approved by the Animal Ethics Committee of Zhejiang Academy of Agricultural Sciences (2023ZAASLA77).

### 2.2 Bone structure analysis and blood chemistry assessment

Distal femur samples preserved in 4% paraformaldehyde were analyzed using SkyScan1176 Micro-CT (Bruker Corporation, Germany). Whole blood samples underwent centrifugation at 2,400 rpm and 4 °C for 15 min. The serum levels of tartrate-resistant acid phosphatase (TRAP), C-terminal telopeptide of type I collagen (CTX-1), interleukin-6 (IL-6), tumor necrosis factor-alpha (TNF-α), and estradiol (E2), and osteoprotegerin (OPG) were quantified using enzyme-linked immunosorbent assay kits provided by servicebio (Wuhan, China). Methodological details are described in Supplementary Material.

### 2.3 Colon histology and short-chain fatty acid analysis

Colon samples were fixed in 4% paraformaldehyde, followed by trimming, dehydration, wax immersion, and paraffin embedding. Hematoxylin and eosin staining was performed. Fecal matter was dissolved in 1:10 phosphate-buffered saline solution and thoroughly mixed. After centrifugation, the supernatant was filtered for SCFAs analysis on a GC-2010 Plus gas chromatograph (Shimadzu Corporation, Kyoto, Japan) equipped with a DB-FFAP column (Agilent Technologies, Santa Clara, CA, United States). Methodological details have been described elsewhere ((Zhao et al., 2023)).

### 2.4 Fecal microbiota analysis

Fecal samples, preserved at −80°C, underwent 16S rDNA sequencing focusing on the V3-V4 region with primers 341F (5′-CCTACGGGNGGCWGCAG-3′) and 805R (5′-GACTACHVGGGTATCTAATCC-3′). The Illumina platform (Kapa Biosciences, Woburn, MA, United States) was used for sequencing, followed by bioinformatics analysis using the Lianchuan Biotechnology platform. Methodological details are described in Supplementary Material.

### 2.5 Serum/fecal untargeted metabolomic analysis

LC-MS analysis of fecal and serum samples, stored at −80°C, was performed using a Vanquish UHPLC System (Thermo Fisher Scientific, United States). Chromatographic separation was conducted on an ACQUITY UPLC^®^ HSS T3 column (150 mm × 2.1 mm, 1.8 µm, Waters, Milford, MA, United States), maintained at 40 °C. The flow rate and injection volume were 0.25 mL/min and 2 μL, respectively. The details of the procedure are provided in Supplementary Material.

### 2.6 Statistical analysis

Data are presented as mean ± standard error of the mean. Analysis of variance (ANOVA) was performed using GraphPad Prism 9.0 (San Diego, CA, United States), and Spearman correlation coefficients were used to ascertain associations between variables. Significance levels are indicated as follows: *p < 0.05 (*), **p < 0.01 (**), ***p < 0.001 (***), and ****p < 0.0001 (****).

## 3 Results

### 3.1 Evaluation of bone metabolism-related parameters in DMY-treated OVX mice

**FIGURE 1:**
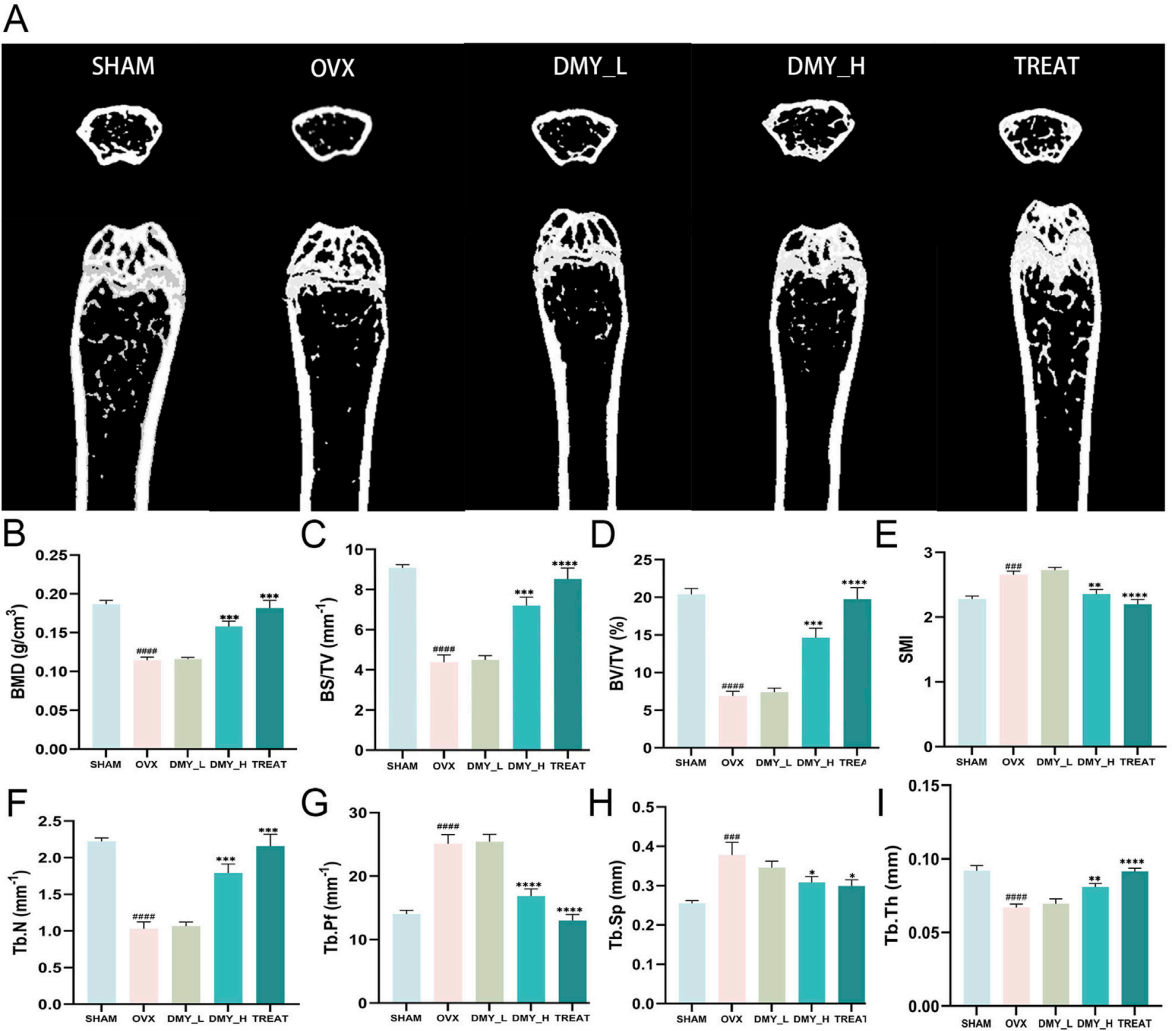
Evaluation of bone metabolism-related parameters in DMY-treated OVX mice **(A)** Representative micro-CT images of femur cross-sections from SHAM, OVX, DMY_L, DMY_H, and TREAT groups. **(B)** Bone mineral density (BMD). **(C)** Bone surface to volume ratio (BS/TV). **(D)** Bone volume to total volume ratio (BV/TV). **(E)** Structural model index (SMI). **(F)** total number of bone trabeculae (Tb.N). **(G)** Trabecular pattern factor (Tb.Pf). **(H)** Trabecular separation (Tb.Sp). **(I)** Trabecular thickness (Tb.Th). Each group consisted of n = 10 mice. Data are expressed as mean ± SEM. Significant differences compared to the SHAM group are indicated by hashtags: ####*p* < 0.0001, ###*p* < 0.001. Significant differences compared to the OVX group are indicated by asterisks: ****p < 0.0001, ***p < 0.001, **p < 0.01, *p < 0.05.

Following an 8-week gavage period, femoral bone microstructure was evaluated using micro-computed tomography (CT) scanning in all groups. Relative to the SHAM group, the OVX group exhibited a significant decrease in trabecular structure, validating the efficacy of the ovariectomy procedure. Notably, the DMY_H and TREAT groups demonstrated substantial increases in trabecular structure, whereas alterations in the DMY_L group were less pronounced (Figure 1A). Compared to the OVX group, the DMY_H group showed marked improvements in bone mineral density (BMD) (Figure 1B), bone surface to volume ratio (Figure 1C), bone volume to tissue volume ratio (Figure 1D), trabecular number (Figure 1F), and trabecular thickness (Figure 1I). In contrast, this enhancement in the DMY_L group was less evident. Regarding the structural model index (Figure 1E), trabecular pattern factor (Figure 1G), and trabecular separation (Figure 1H), the values in the DMY_H group were lower than those in the OVX group, whereas the decreases observed in the DMY_L group were minimal. These findings indicate that high-dose DMY substantially mitigated osteoporosis markers induced by ovariectomy, whereas low-dose DMY had a less pronounced effect.

### 3.2 DMY mitigates histopathological damage in the colon and regulates SCFAs production in OVX mice

**FIGURE 2:**
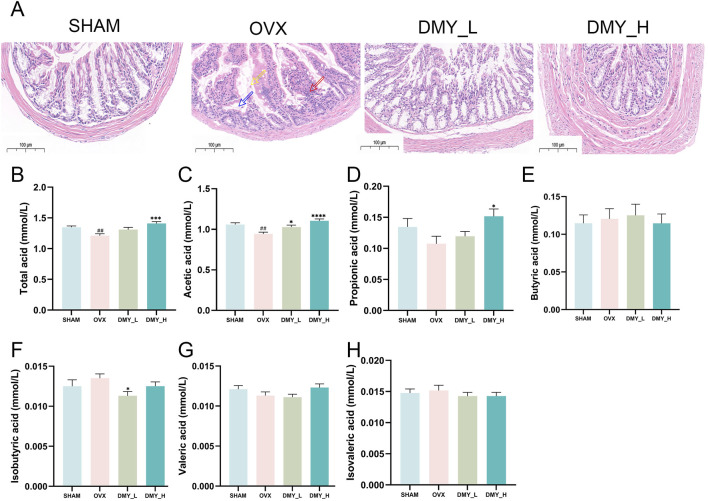
DMY mitigates histopathological damage in the colon and regulates SCFAs production in OVX mice **(A)** Representative H&E-stained colon sections from SHAM, OVX, DMY_L, and DMY_H groups. **(B)**, Total SCFA. **(C)** acetic acid. **(D)** Propionic acid. **(E)** Butyric acid. **(F)** Isobutyric acid. **(G)** Valeric acid. **(H)** Isovaleric acid. Each group consisted of n = 10 mice. Data are expressed as mean ± SEM. Significant differences compared to the SHAM group are indicated by hashtags: ##*p* < 0.01. Significant differences compared to the OVX group are indicated by asterisks: ****p < 0.0001, ***p < 0.001, *p < 0.05.

Hematoxylin and eosin-stained colon tissue sections from the SHAM, OVX, DMY_L, and DMY_H groups are shown in Figure 2A. The SHAM group showed intact colonic mucosa with well-organized glandular cells. OVX mice exhibited glandular epithelial cell shedding (indicated by yellow arrows), inflammatory cell infiltration, crypt destruction (red arrows), and submucosal edema (blue arrows), which were markedly diminished in both the DMY_L and DMY_H groups. Gas chromatographic analysis of SCFAs revealed significant alterations in fecal SCFA production. The concentrations of total acids (Figure 2B), acetic acid (Figure 2C), and propionate acid (Figure 2D) were significantly higher in the DMY_H group than in the OVX group (***p < 0.001 and **p < 0.05, respectively). The concentrations of butyric acid (Figure 2E), isobutyric acid (Figure 2F), and valeric acid (Figure 2G) were not significantly different among the groups, whereas isovaleric acid (Figure 2H) levels were consistent in all groups. These findings indicate that DMY, especially at higher doses, may alleviate histological damage in the colon and modulate SCFA production.

### 3.3 DMY enhances bone metabolic marker levels and reduces inflammatory factor levels in OVX mice, with correlation matrix analysis including BMD, SCFAS, and serum biomarkers

**FIGURE 3:**
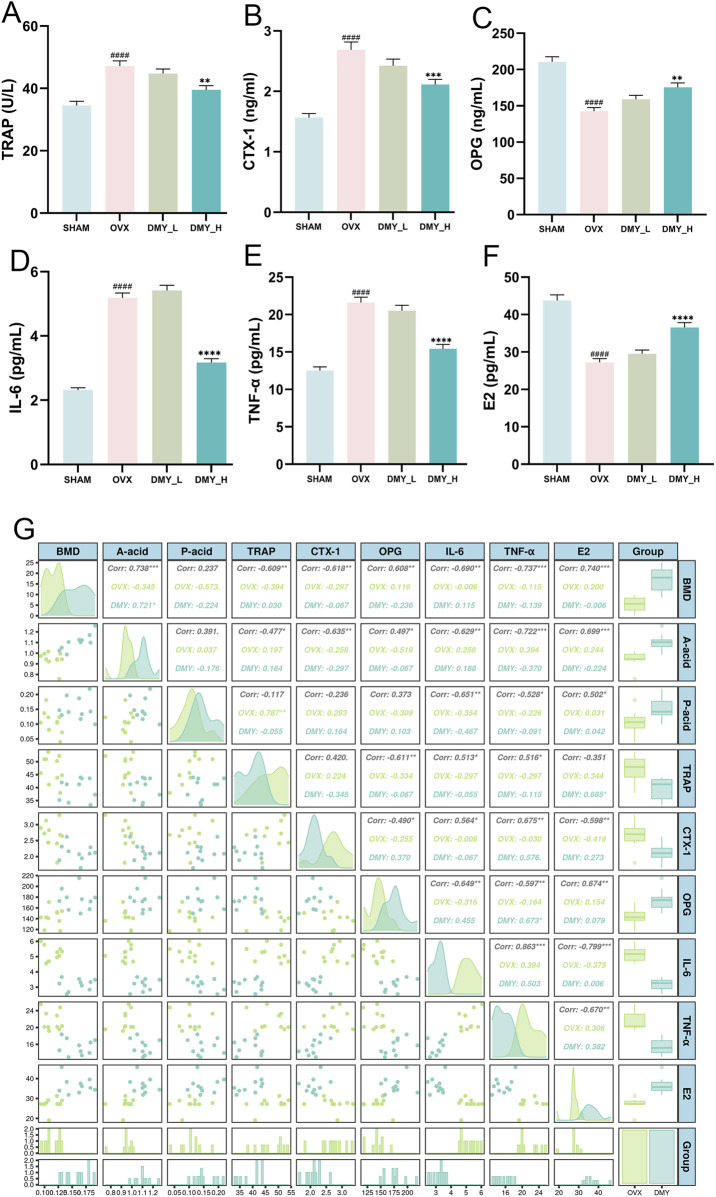
DMY enhances bone metabolic marker levels and reduces inflammatory factor levels in OVX mice, with correlation matrix analysis including BMD, SCFAS, and serum biomarkers **(A)** Tartrate-Resistant Acid Phosphatase (TRAP). **(B)** C-Terminal Telopeptide of Type I Collagen (CTX-1). **(C)** Osteoprotegerin (OPG). **(D)** Interleukin 6 (IL-6). **(E)** Tumor Necrosis Factor Alpha (TNF-α). **(F)** Estradiol (E2). **(G)** The correlation matrix showcases the relationships between BMD, SCFAs (acetic acid and propionic acids), and serum markers (TRAP, CTX-1, OPG, IL-6, TNF-α, E2). Each group consisted of n = 10 mice, with data expressed as mean ± SEM. Hashes denote significant differences compared to the SHAM group (####*p* < 0.0001), while asterisks indicate differences compared to the OVX group (****p < 0.0001, ***p < 0.001, **p < 0.01).

The OVX mice showed significantly elevated TRAP levels, indicating increased bone resorption, which was mitigated by high-dose DMY treatment (Figure 3A). The DMY_H group showed a notable decrease in serum levels of CTX-1, compared to those in the OVX mice, suggesting diminished bone turnover (Figure 3B). OPG concentrations in the OVX group were significantly lower than those in the SHAM group but were restored in the DMY_H group (Figure 3C). IL-6 (Figure 3D), TNF-α (Figure 3E), and E2 (Figure 3F) levels declined in the DMY_H group, compared to those in the OVX group, suggesting some anti-inflammatory properties of high-dose DMY. Correlation analysis revealed associations between BMD and serum biomarkers (Figure 3G). BMD strongly and negatively correlated with TRAP (Corr. −0.609**) and positively correlated with E2 (Corr = 0.740**). Serum IL-6 levels negatively correlated with acetate (Corr. −0.629**) and propionate (Corr. −0.722**) levels. These findings indicate that high-dose DMY may protect bone metabolism, potentially by modulating osteoclast activity, inflammatory levels, and SCFA production.

### 3.4 Influence of DMY on gut microbiota composition in OVX mice

**FIGURE 4:**
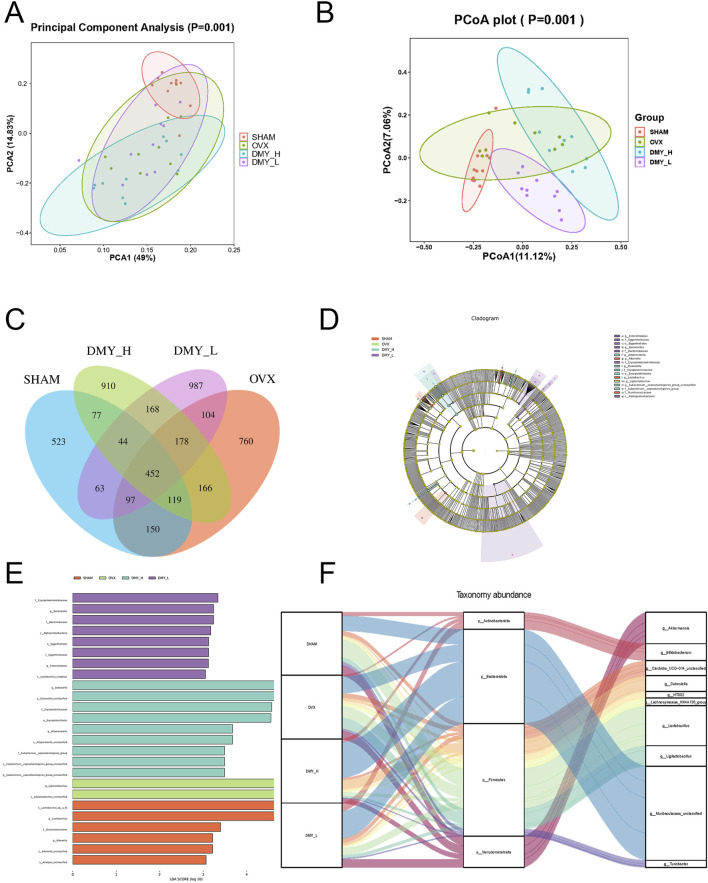
Influence of DMY on gut microbiota composition in OVX mice **(A)** Principal Component Analysis (PCA). **(B)** Principal Coordinates Analysis (PCoA). **(C)** Venn diagram of OTUs. **(D, E)** LEfSe analysis. **(F)** Sankey diagram. Each group consisted of n = 10 mice.

Principal component analysis and principal coordinate analysis were used to assess the impact of DMY treatment on the gut microbiota structure. The principal component analysis results (Figure 4A) showed clear clustering among the SHAM, OVX, DMY_L, and DMY_H groups, suggesting that DMY treatment significantly influenced the overall composition of the gut microbiota (*p* = 0.001). The principal coordinate analysis outcomes (Figure 4B) corroborated these observations, showing marked differences among the experimental groups (*p* = 0.001), thus highlighting DMY effects on the microbial community structure. The Venn diagrams (Figure 4C) depict unique and shared operational taxonomic units across the SHAM, OVX, and DMY-treated groups. LEfSe analysis (Figures 4D, E) revealed distinct gut microbial compositions in each group, with specific dominant bacterial taxa. Notably, after filtering (*p* < 0.05, LDA = 3), the SHAM group was characterized mainly by Rikenella and *Lactobacillus*, the OVX group by Ligilactobacillus, the DMY_L group by *Bacteroides* and Enterorhabdus, and the DMY_H group by Dubosiella, Alloprevotella, and Eubacterium__coprostanoligenes_group_unclassified post-high-dose DMY intervention. The Sankey diagram (Figure 4F) revealed the relative abundances and variations at the phylum and genus levels among the four groups, indicating that DMY intervention induced alterations in the intestinal microbial community of the OVX mice. These changes in the gut microbiota composition may constitute one of the mechanisms by which DMY effectively treats postmenopausal osteoporosis.

### 3.5 Impact of DMY on the gut microbiota composition at phylum and genus levels in OVX mice

**FIGURE 5:**
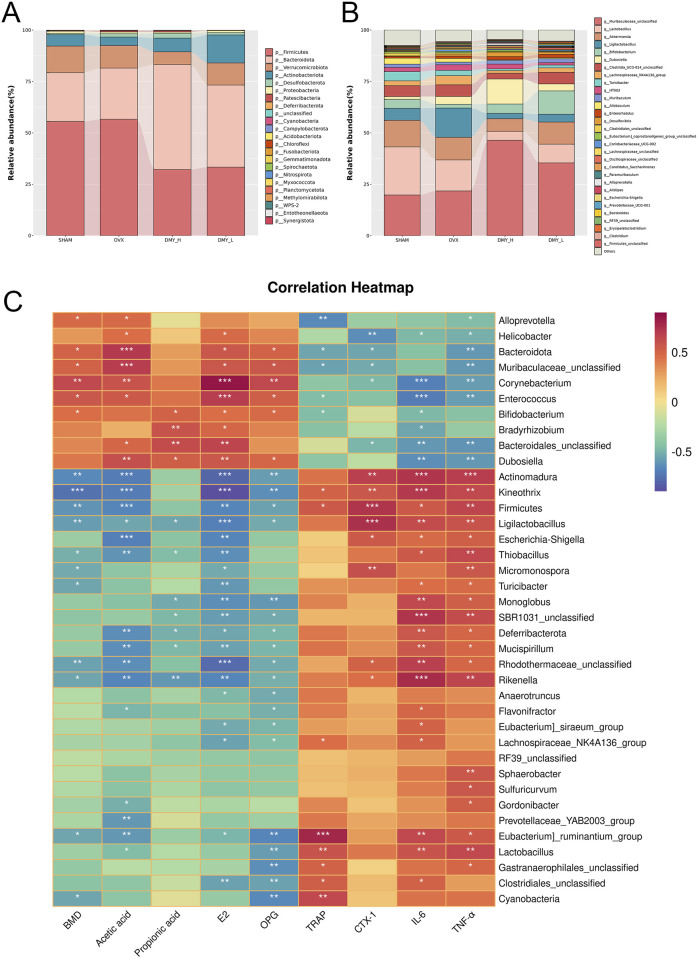
Impact of DMY on the gut microbiota composition at phylum and genus levels in OVX mice **(A)** community structures of the observed samples at the phylum levels **(B)** community structures of the observed samples at the genus levels. **(C)** Spearman correlation heatmap. Warm colors for positive and cool colors for negative correlations. Asterisks denote the level of statistical significance, with *p < 0.05, **p < 0.01, ***p < 0.001.

We investigated the variations in intestinal microbiota richness by analyzing both the phylum and genus levels. Firmicutes and Bacteroidetes were the predominant phyla, constituting over 75% of the total relative abundance in all four groups (Figure 5A). The DMY_H group exhibited a notable decrease in Firmicutes, Deferribacterota, and Cyanobacteria and an increase in Bacteroidetes abundance, compared to those in the OVX group (Supplementary Figure S1A). Distinct disparities in the gut microbiota structure at the genus level were observed among the groups (Figure 5B), with the DMY_H group showing significant divergence in 34 genera from the OVX group (Supplementary Figure S1B). A subsequent correlation analysis was conducted between 38 different microbial taxa at the phylum and genus levels in the feces samples of the DMY_H and OVX groups and various serum biomarkers related to bone metabolism, inflammation, and SCFA synthesis (Figure 5C). The presence of certain microbes was linked with multiple biomarker levels. For example, Actinomadura showed a positive correlation with CTX-1, IL-6, TNF-α, and a negative correlation with BMD, acetate, E2, and OPG levels. Similar associations were noted for taxa such as Kineothrix, Firmicutes, Ligilactobacillus, Rikenella, and Eubacteriumruminantium. In contrast, taxa such as Corynebacterium, Bacteroidota, and *Enterococcus* negatively correlated with TRAP, CTX-1, IL-6, TNF-α, and positively with BMD, acetate, propionate, E2, and OPG levels. These correlations underscore the complex interactions between gut microbiota and host physiology, indicating that high-dose DMY may modulate biomarkers associated with bone metabolism, inflammation, and SCFA synthesis through alterations in the gut microbiota composition, thereby contributing to osteoporosis amelioration.

### 3.6 Serum metabolomic analysis identifying metabolic shifts in OVX mice post-high-dose DMY intervention

**FIGURE 6:**
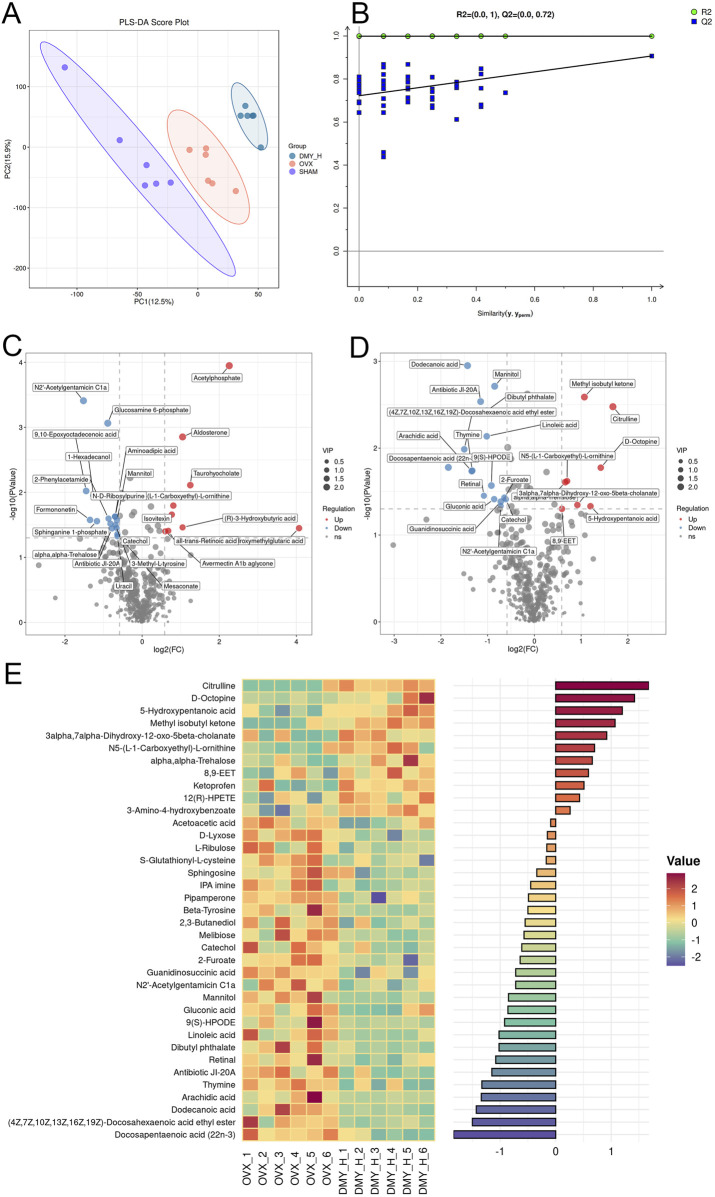
Serum metabolomic analysis identifying metabolic shifts in OVX mice post-high-dose DMY intervention **(A)** Partial Least Squares-Discriiminate Analysis (PLS-DA). **(B)** Permutation test. **(C)** Volcano plots of the differential metabolites between SHAM and OVX. **(D)** Volcano plots of the differential metabolites between DMY_H and OVX. **(E)** The heatmap of abundance differences of metabolites between DMY_H and OVX. Warm colors for positive and cool colors for negative correlations. Each group consisted of n = 6 mice.

Partial least squares discriminant analysis (PLS-DA) demonstrated distinct metabolomic profiles among the SHAM, OVX, and DMY_H groups, signifying specific serum metabolic changes in the OVX mice after high-dose DMY administration (Figure 6A). The permutation test results of the PLS-DA model showed that R2Y, the explanatory power of the model’s dependent variables, and Q2, the model’s predictive accuracy, were both above 0.5, confirming its predictive capacity (Figure 6B). Volcano plots illustrated the differential metabolites between the SHAM and OVX groups, as well as between the DMY_H and OVX groups, underscoring the significantly modulated metabolites (Figures 6C, D). Compared to the SHAM group, the OVX group had 24 metabolites that were upregulated and 15 that were downregulated (Supplementary Figure S2A). Meanwhile, the DMY_H group had 11 upregulated and 26 downregulated metabolites, compared to the OVX group. These 37 metabolites were subjected to correlation analysis based on their grouping (Figure 6E). A heatmap bar graph captured the varying differential metabolites and their expression levels between the DMY_H and OVX groups. Notably, citrulline, D-octopine, and 5-hydroxypentanoic acid levels, among others, were elevated in the DMY_H group, whereas catechol, sphingosine, linoleic acid, and arachidic acid levels were reduced.

### 3.7 Fecal metabolomic profiling reflecting metabolic shifts in OVX mice post-high-dose DMY treatment

**FIGURE 7:**
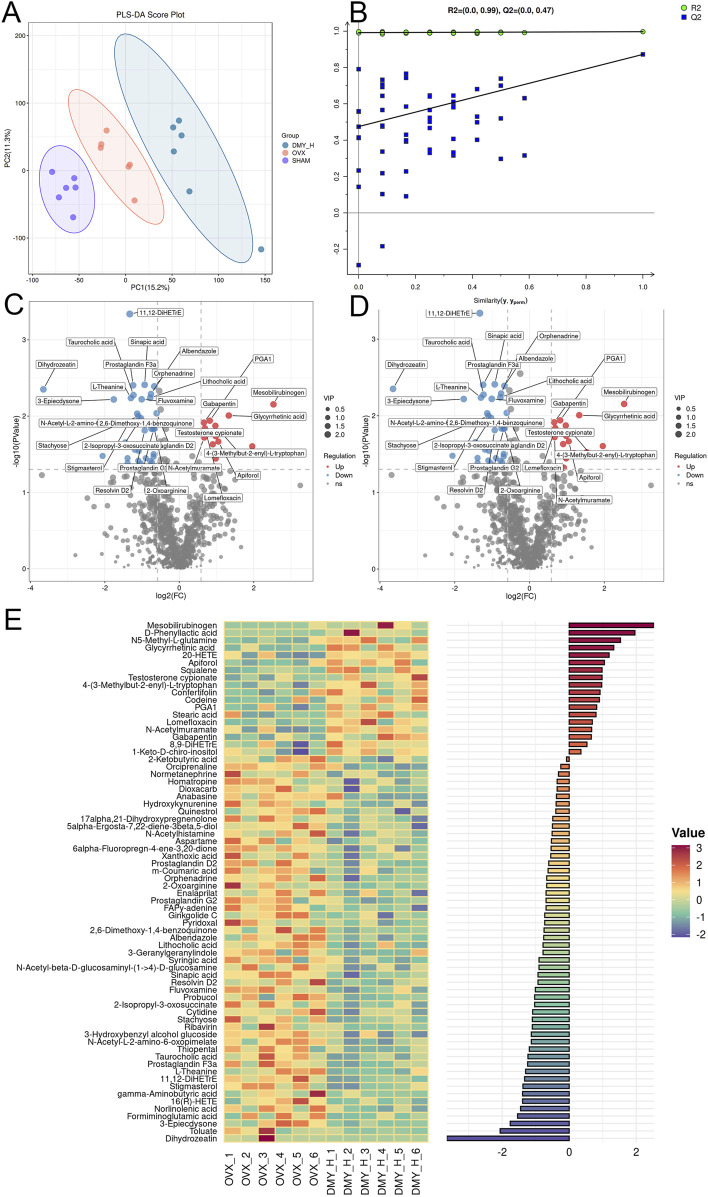
Fecal metabolomic profiling reflecting metabolic shifts in OVX mice post-high-dose DMY treatment Fecal metabolomics results of OVX mice after high-dose DMY intervention. **(A)** Partial Least Squares-Discriiminate Analysis (PLS-DA). **(B)** Permutation test. **(C)** Volcano plots of the differential metabolites between SHAM and OVX. **(D)** Volcano plots of the differential metabolites between DMY_H and OVX. **(E)** The heatmap of abundance differences of metabolites between DMY_H and OVX. Warm colors for positive and cool colors for negative correlations. Each group consisted of n = 6 mice.

PLS-DA indicated disparities in metabolite composition among the SHAM, OVX, and DMY_H groups, suggesting distinctive fecal metabolomic changes in the OVX mice following high-dose DMY administration (Figure 7A). Permutation test results of the PLS-DA model confirmed its strong predictive accuracy (Figure 7B). Volcano plots elucidated the differential metabolites between the SHAM and OVX groups and between the DMY_H and OVX groups, emphasizing the modulated metabolites (Figures 7C, D). Compared to the SHAM group, the OVX group exhibited nine upregulated and 42 downregulated metabolites (Supplementary Figure S2B). After high-dose DMY intervention, the DMY_H group demonstrated 18 upregulated metabolites, including glycyrrhetinic acid, testosterone cypionate, and stearic acid, and 52 downregulated metabolites, such as lithocholic acid, hydroxykynurenine, stigmasterol, and gamma-aminobutyric acid, compared to the OVX group. We performed a correlation analysis of these 70 metabolites according to their respective groups (Figure 7E), with a heatmap bar graph presenting the distinct differential metabolites and their expression levels between the DMY_H and OVX groups.

### 3.8 Comprehensive analysis of metabolomics, microbiomics, and biomarkers

**FIGURE 8:**
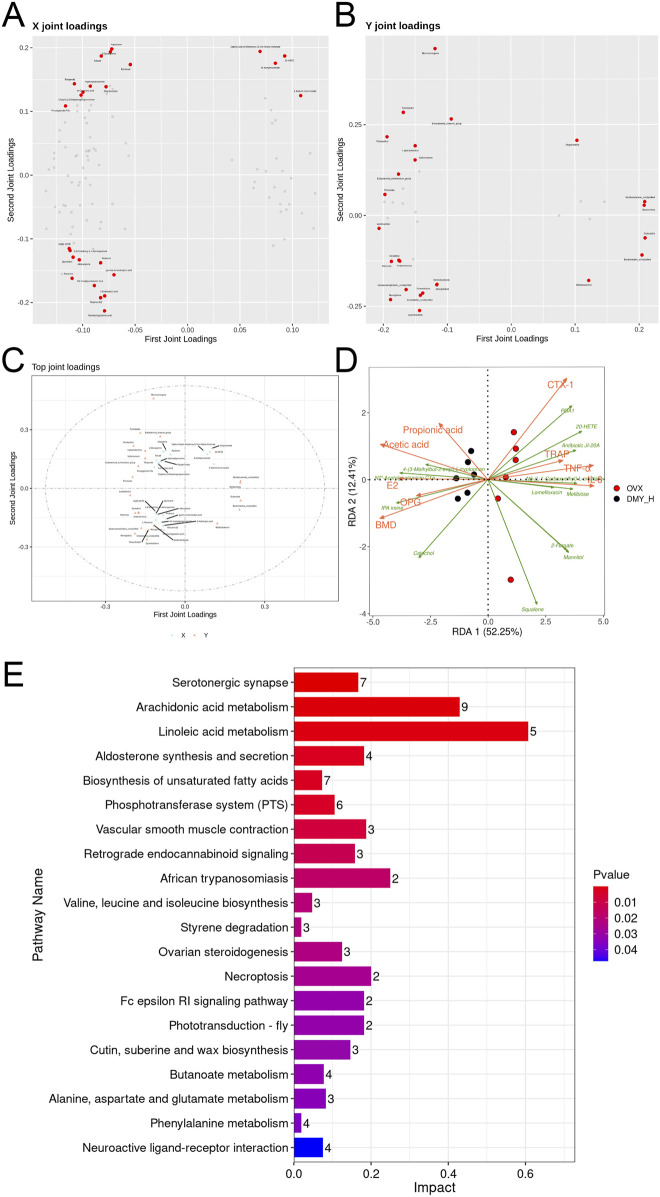
Comprehensive analysis of metabolomics, microbiomics, and biomarkers **(A)** O2PLS loading plots of metabolites. **(B)** O2PLS loading plots of bacteria. **(C)** O2PLS joint loadings plot of the top 25 contributing factors from both the metabolites and bacteria. **(D)** Redundancy Analysis (RDA). **(E)** The KEGG pathway enrichment scatter plot of the 107 differential metabolites from both serum and feces between DMY_H and OVX group.

Two-way orthogonal PLS was used for multivariate analysis of the combined metabolomic and microbiomic data, with loading plots demonstrating the contributions of specific gut microbiota and metabolites (Figures 8A, B). The direction and magnitude of the loading values indicated either a positive or a negative correlation with other omics data, with larger absolute values indicating stronger associations. Forminoglutamic acid, aspartame, two-oxoarginine, resolvin D2, and 2-ketobutyric acid levels were significantly correlated with gut microbiota composition. Similarly, Dubosiella, Muribaculaceae_unclassified, Bacteroidetes, *Lactobacillus*, and Bacteroidales_unclassified showed notable correlations (Figure 8C). We examined the common differential metabolites that were consistently regulated in both serum and fecal samples (SupplementaryTable S1). Thirteen selected metabolites were analyzed using redundancy analysis, along with bone metabolism marker, inflammatory factor, and SCFA levels. The associations among metabolites with environmental factors were inferred from the vector angles and projection positions, revealing strong links of PGA1, 20-HETE, and antibiotic JI-20A with CTX-1, TRAP, TNF-α, and IL-6, and close relations of 4-(3-methylbut-2-enyl)-L-tryptophan, N2′-acetylgentamicin C1a, and IPA imine with BMD, E2, OPG, acetate, and propionate levels (Figure 8D). Pathway enrichment analysis of significantly altered metabolites post-high-dose DMY intervention showed 23 distinct pathways (Figure 8E), including serotonergic synapse, arachidonic acid metabolism, aldosterone synthesis and secretion, and the biosynthesis of unsaturated fatty acid pathways, which may be the mechanisms of DMY effects on bone metabolism.

## 4 Discussion

DMY is a flavonoid compound, which may benefit human health (Wen et al., 2024; Li et al., 2023). In this study, we have shown that orally administered high-dose DMY may ameliorate osteoporosis symptoms in the OVX mice. These findings are consistent with imaging studies and serum bone metabolism marker analyses. Overall, this evidence suggests that DMY may help improve outcomes in osteoporosis.

In the OVX mouse model, the primary source of estrogen is removed, leading to a notable decrease in blood E2 levels. E2 protects the intestine, helping maintain intestinal barrier integrity, regulate immune responses, and suppress inflammation (Shieh et al., 2020). Reduced E2 levels result in increased intestinal mucosal permeability, which initiates localized inflammatory responses and tissue damage, that may lead to systemic inflammation. The absence of E2 has been linked to increased IL-6 and TNF-α levels, which, by activating the RANKL expression and the RANK/RANKL and TNF-α/NF-κB signaling pathways, foster osteoclast precursor differentiation and maturation, thereby expediting bone resorption and loss (Hu et al., 2022; Pacifici, 2012). The concurrent increases in IL-6 and TNF-α levels impede osteoblast activity, diminishing new bone formation. Our findings demonstrate that DMY elevates E2 levels and reduces IL-6 and TNF-α levels in the serum of the OVX mice. Furthermore, DMY increased the production of SCFAs (acetate and propionic acid) in the intestine, which is consistent with the findings of Zhang et al., where fecal microbiota transplantation from healthy mice increased the acetate and propionate levels in the OVX mice (Zhang et al., 2022). SCFAs, that are gut microbiota metabolic byproducts derived from anaerobic carbohydrate fermentation, affect immune modulation, tumor suppression, and diabetes progression. Some evidence suggests that propionate, through metabolic reprogramming, enhances the glycolysis of osteoclast precursors while reducing oxidative phosphorylation, thereby inhibiting osteoclast differentiation pathways (Lucas et al., 2018). A recent study has shown that SCFA supplementation ameliorates osteoporosis progression in psoriatic arthritis by inhibiting osteoclast differentiation (Yang et al., 2024). Herein, we have shown a significant positive correlation between the intestinal SCFA synthesis rate (notably acetate and propionate acid) and bone protective indicator levels (E2, OPG, and BMD), and a negative correlation with bone loss indicator levels (IL-6, TNF-α, TRAP, and CTX-1). This evidence suggests that DMY modulates the gut microbiota, thereby helping correct the gut-bone axis imbalance in the OVX mice.

The gut microbiota may be a target in osteoporosis treatment (Lorenzo, 2021; Seely et al., 2021). Guo et al. have shown that *Lactobacillus rhamnosus GG* may enhance bone microstructure and metabolic indices in the OVX rats by modulating the Th17/Treg balance, while altering the Firmicutes/Bacteroidota ratio increased by OVX, providing a candidate mechanism for the gut microbiota affecting osteoporosis (Guo et al., 2023). Herein, DMY administration reduced Firmicutes abundance (56.63%–32.15%) and increased Bacteroidota abundance (24.88%–51.10%). Alongside the phylum-level changes, DMY markedly altered the abundance of various genera, such as Muribaculaceae_unclassified, Dubosiella, Bifidobacterium, Alloprevotella, *Helicobacter*, *Enterococcus*, Bacteroidales_unclassified, Corynebacterium, and Bradyrhizobium, which were increased in the treated compared to those in the OVX mice. Similar to the effects of glycyrrhiza polysaccharides on immunosuppressed mice, increasing Muribaculaceae_unclassified and boosting intestinal acetate and propionate production (Song et al., 2023), DMY may influence SCFA production, mitigate inflammation, and exert anti-osteoporotic effects. Zhu et al. examined *Tilapia nilotica* fish head lipid effects on OVX rats, showing similar effects to those described herein, whereby fish head lipids increased Dubosiella abundance and decreased TNF-α and IL-6 levels (Zhu et al., 2021). This evidence suggests that DMY may affect systemic inflammation levels and bone loss by regulating the gut microbiota in the OVX mice.

Bifidobacterium suppresses osteoclast activity and produces anti-osteoporotic effects via SCFAs, equol (Eq), exopolysaccharides, and by inhibiting the TNF-α/NF-κB and RANKL/RANK/OPG pathways (Wu et al., 2023). Our study noted an increase in Bifidobacterium from 1.84% to 4.53% after DMY intervention. Other significantly enriched genera, such as Alloprevotella, demonstrated similar osteoprotective effects (Liu et al., 2020; Tu et al., 2020). The anti-osteoporotic function of *Enterococcus* may be linked to aromatic amine production, which stimulates colonic serotonin production (Sugiyama et al., 2022). Despite their lower relative abundances, the contributions of these genera to osteoporosis require further research.

In addition to modulating the gut microbiota, DMY altered the serum and fecal metabolite compositions in the OVX mice. The serum comprises nearly a thousand endogenous small molecule metabolites with complex compositions and varied polarities, including growth factors, inorganic ions, amino acids, nucleosides, lipids, and sterols. Investigating serum metabolite alterations helps capture changes in the body physiological and pathological states and may inform disease diagnosis and treatment. Similarly, fecal metabolites are indicative of metabolic processes and closely associated with the gut microbiota, host genetics, and disease phenotypes. They are the final products of intestinal digestion and are easily accessible and vital for studying the effects of drugs, diet, and various interventions on the body. In this study, DMY affected serum and fecal metabolite concentrations, suggesting its systemic effects in the OVX mice. The KEGG enrichment analysis of all differential metabolites identified 23 significantly enriched pathways, directly and indirectly linked to osteoporosis onset and treatment. Notably, the arachidonic acid metabolism pathway emerged as a potential target, given its enrichment with differential metabolites. In previous studies, natural plant compounds, such as Acanthopanax senticosus and osthole, improve osteoporosis markers in the OVX mice by modulating this pathway (Zhang et al., 2019; Si et al., 2020). Li et al. reported the efficacy of puerarin in osteoporosis therapy, linked to the reduction in inflammatory factor release, modulated by unsaturated fatty acid production, specifically the n-6/n-3 polyunsaturated fatty acid ratio (Li et al., 2022). In our study, metabolites such as linoleic acid, arachidonic acid, and gamma-linolenic acid underwent significant modulation, suggesting DMY efficacy in the OVX mice. The serum and fecal metabolites examined showed consistent patterns of down- and upregulation in the SHAM and DMY groups, compared to those in the OVX group. The upregulated metabolites included catechol, 4-(3-methylbut-2-enyl)-L-tryptophan, IPA imine, and serum N2′-acetylgentamicin C1a, and downregulated metabolites included squalene, lomefloxacin, N5-(L-1-carboxyethyl)-L-ornithine, antibiotic JI-20A, 2-furoate, mannitol, and 20-HETE. Although little is known about these metabolites in the context of bone metabolism, the present study indicates their potential involvement in oxidative stress and SCFAs metabolism, which may be future targets for osteoporosis treatment.

This study had some limitations. First, the concentration range used was relatively narrow; future studies should evaluate the specific dose-response relationships involved to determine the optimal therapeutic dosage. Second, this study was a correlation-based study, and determining causal relationships was outside the study scope; future studies should evaluate the causal relationships among the parameters described. Further studies may help elucidate the effects of DMY on osteoporosis development, helping accumulate the evidence required before commencing translational research.

## 5 Conclusion

In summary, this study examined the effects of DMY on osteoporosis in the OVX mice by evaluating the serum biomarker levels, gut microbiota composition, and serum and fecal metabolite levels. The findings of this study suggest that DMY may affect osteoporosis in the OVX mice by reducing inflammation levels and mitigating colonic injury. DMY may alter the gut microbiota composition and enhance the production of intestinal SCFAs, notably acetate and propionate, as well as change the serum and fecal metabolite profile in the mice. The affected metabolites include 5-hydroxypentanoic acid, linoleic acid, arachidic acid, glycyrrhetinic acid, and gamma-aminobutyric acid, which are involved in biological pathways associated with bone metabolism. Overall, these findings suggest that DMY may have anti-osteoporotic properties, mediated by the gut-bone axis signaling, contributing to the development of the associated theories.

## Data Availability

The sequences obtained in the study were deposited in the NCBI Sequence Read Archive under accession no. PRJNA1163031. Additional data sources are also available from the corresponding authors upon reasonable request.

## Abbreviations

CT, computed tomography; CTX-1, C-terminal telopeptide of type I collagen; DMY, dihydromyricetin; DMY_L, low-dose DMY group; DMY_H, high-dose DMY group; E2, estradiol; IL-6, interleukin-6; OPG, osteoprotegerin; OVX, ovariectomized; SCFA, short-chain fatty acid; SHAM, sham group; TNF-α, tumor necrosis factor-alpha; TRAP, tartrate-resistant acid phosphatase; TREAT, treatment group.
