# Supplementary material for: Dihydromyricetin ameliorate postmenopausal osteoporosis in ovariectomized mice: Integrative microbiomic and metabolomic analysis
Source: Front Pharmacol. 2024 Oct 2;15:1452921. doi: 10.3389/fphar.2024.1452921 (PMC11479887; doi:10.3389/fphar.2024.1452921)
Supplement: Supplementary file 1 [file DataSheet1.docx]

**Blood Chemistry Assessment**

Blocking: Each well was filled with 200 μL of blocking solution and incubated at 37°C for 1-2 hours.

Washing: The plate was washed 3-5 times using a plate washer or manually by adding washing buffer and soaking, then drying on absorbent paper.

Sample Addition: 100 μL of appropriately diluted samples were added to the wells. Blank, serially diluted standard, negative control, and positive control wells were also prepared.

Incubation: The plate was sealed and incubated at 37°C for 1-2 hours.

Secondary Antibody Addition: 100 μL of diluted biotinylated antibody was added to each well.

Incubation: The plate was sealed and incubated at 37°C for 1 hour.

Enzyme Conjugate Addition: 100 μL of enzyme conjugate solution was added to each well.

Incubation: The plate was sealed and incubated at 37°C in the dark for 30 minutes.

Substrate Addition: 100 μL of TMB substrate solution was added to each well and incubated at 37°C in the dark for 10-30 minutes until a clear color gradient was observed.

Reaction Termination: 100 μL of 2M sulfuric acid was added to each well to stop the reaction, changing the color from blue to yellow.

Measurement: Within 10 minutes, the optical density (OD) at 450 nm was measured using a microplate reader, zeroing with a blank control.

**Fecal Microbiota Analysis**

**Material and method description of 16SrDNA sequencing**

**DNA extractions**

DNA from different samples was extracted using the CTAB according to manufacturer ’s instructions. The reagent which was designed to uncover DNA from trace amounts of sample has been shown to be effective for the preparation of DNA of most bacteria. Nuclear-free water was used for blank. The total DNA was eluted in 50 μL of Elution buffer and storedat -80 °C until measurement in the PCR by LC-Bio Technology Co., Ltd, Hang Zhou, Zhejiang Province, China.

**PCR amplification and 16S rDNA sequencing**

| **Region** | **Primers** |
| --- | --- |
| V3-V4 | 341F (5'-CCTACGGGNGGCWGCAG-3')  805R(5'-GACTACHVGGGTATCTAATCC-3') |

The 5' ends of the primers were tagged with specific barcods per sample and sequencing universal primers.PCR amplification was performed in a total volume of 25 μL reaction mixture containing 25 ng of template DNA, 12.5 μL PCR Premix, 2.5 μL of each primer, and PCR-grade water to adjust the volume. The PCR conditions to amplify the prokaryotic 16S fragments consisted of an initial denaturation at 98 ℃ for 30 seconds; 32cycles of denaturation at 98 ℃ for 10 seconds, annealing at 54℃ for 30 seconds, and extension at 72 ℃ for 45 seconds; and then final extension at 72 ℃ for 10 minutes. The PCR products were confirmed with 2% agarose gel electrophoresis. Throughout the DNA extraction process, ultrapure water, instead of a sample solution, was used to exclude the possibility of false-positive PCR results as a negative control. The PCR products were purifyied by AMPure XT beads (Beckman Coulter Genomics, Danvers, MA, USA) and quantified by Qubit( Invitrogen, USA). The amplicon pools were prepared for sequencing and the size and quantity of the amplicon library were assessed on Agilent 2100 Bioanalyzer (Agilent, USA) and with the Library Quantification Kit for Illumina (Kapa Biosciences, Woburn, MA, USA), respectively. The libraries were sequenced on NovaSeq PE250 platform.

**Data analysis**

Samples were sequenced on an Illumina NovaSeq platform according to the manufacturer's recommendations, provided by LC-Bio. Paired-end reads was assigned to samples based on their unique barcode and truncated by cutting off the barcode and primer sequence. Paired-end reads were merged using FLASH. Quality filtering on the raw reads were performed under specific filtering conditions to obtain the high-quality clean tags according to the fqtrim(v0.94). Chimeric sequences were filtered using Vsearch software(v2.3.4). After dereplication using DADA2,we obtained feature table and feature sequence.Alpha diversity and beta diversity were calculated by normalized to the same sequences randomly.Then according to SILVA(release 138) classifier, feature abundance was normalized using relative abundance of each sample . Alpha diversity is applied in analyzing complexity of species diversity for a sample through 5 indices, including Chao1, Observed species, Goods coverage, Shannon, Simpson, and all this indices in our samples were calculated with QIIME2. Beta diversity were calculated by QIIME2,the graphs were drew by R package.Blast was used for sequence alignment, and the feature sequences were annotated with SILVA database for each representative sequence.Other diagrams were implemented using the R package(v3.5.2).

**Serum/Fecal Untargeted Metabolomic Analysis**

**Chemicals and reagents**

LC-MS grade methanol (MeOH) was purchased from Fisher Scientific (Loughborough, UK). 2-Amino-3-(2-chloro-phenyl)-propionic acid was obtained from Aladdin (Shanghai, China).

**Instruments**

High speed freezing centrifuge was obtained from Hunan Xiangyi Experiment Equipment Co., Ltd. (Hunan, China). Vortex mixer was obtained from Haimen Kylin-bell Lab Instruments Co., Ltd. (Haimen, China). Ultrasonic cleaner was obtained from Kunshan Shumei Experiment Equipment Co., Ltd. (Kunshan, China). Tissue grinder was obtained from Zhejiang Meibi Experiment Equipment Co., Ltd. (Zhejiang, China). Microporous membrane filters (0.22 µm) was purchased from Tianjin Jinteng Experiment Equipment Co., Ltd. (Tianjin, China). Glass bead was obtained from Sigma-Aldrich (Shanghai, China).

**Sample preparation**

1. Accurately weigh an appropriate amount of sample into a 2 mL centrifuge tube, add 600 µL MeOH (stored at -20℃) (Containing 2-Amino-3-(2-chloro-phenyl)-propionic acid (4 ppm)， vortex for 30 s;

2. Add 100 mg glass bead, placed in a tissue grinder for 90 s at 60 Hz;

3. Room temperature ultrasound for 10 min;

4. Centrifuge for 10 min at 12,000 rpm and 4℃, filter the supernatant by 0.22 μm membrane and transfer into the detection bottle for LC-MS detection.

**Reagents**

LC-MS grade acetonitrile (ACN) was purchased from Fisher Scientific (Loughborough, UK). Formic acid was obtained from TCI (Shanghai, China). Ammonium formate was obtained from Sigma-Aldrich (Shanghai, China). Ultrapure water was generated using a Milli-Q system (Millipore, Bedford, USA).

**Liquid chromatography conditions**

The LC analysis was performed on a Vanquish UHPLC System (Thermo Fisher Scientific, USA). Chromatography was carried out with an ACQUITY UPLC ® HSS T3 (150 × 2.1 mm, 1.8 µm) (Waters, Milford, MA, USA). The column maintained at 40 ℃. The flow rate and injection volume were set at 0.25 mL/min and 2 μL, respectively. For LC-ESI (+)-MS analysis, the mobile phases consisted of (B2) 0.1% formic acid in acetonitrile (v/v) and (A2) 0.1% formic acid in water (v/v). Separation was conducted under the following gradient: 0~1 min, 2% B2; 1~9 min, 2%~50% B2; 9~12 min, 50%~98% B2; 12~13.5 min, 98% B2; 13.5~14 min, 98%~2% B2; 14~20 min, 2% B2. For LC-ESI (-)-MS analysis, the analytes was carried out with (B3) acetonitrile and (A3) ammonium formate (5mM). Separation was conducted under the following gradient: 0~1 min, 2% B3; 1~9 min, 2%~50% B3; 9~12 min, 50%~98% B3; 12~13.5 min, 98% B3; 13.5~14 min, 98%~2% B3; 14~17 min, 2% B3.

**Mass spectrum conditions**

Mass spectrometric detection of metabolites was performed on Orbitrap Exploris 120 (Thermo Fisher Scientific, USA) with ESI ion source. Simultaneous MS1 and MS/MS (Full MS-ddMS2 mode, data-dependent MS/MS) acquisition was used. The parameters were as follows: sheath gas pressure, 30 arb; aux gas flow, 10 arb; spray voltage, 3.50 kV and -2.50 kV for ESI(+) and ESI(-), respectively; capillary temperature, 325 ℃ ; MS1 range, m/z 100-1000; MS1 resolving power, 60000 FWHM; number of data dependant scans per cycle, 4; MS/MS resolving power, 15000 FWHM; normalized collision energy, 30%; dynamic exclusion time, automatic.


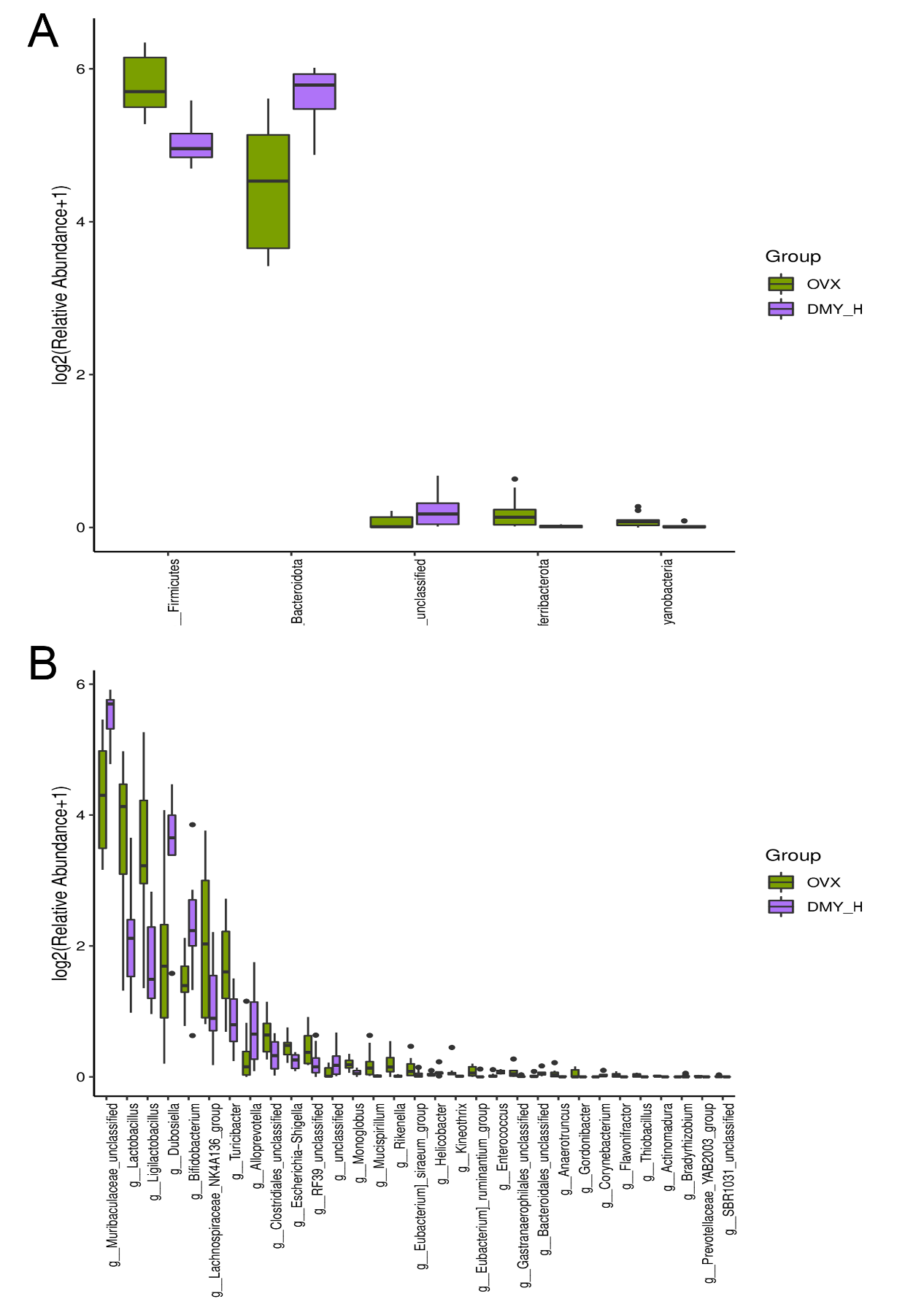


Figure S1 Differential microbial communities at the phylum and genus levels between DMY_H and OVX groups. (A) Phylum. (B) Genus.
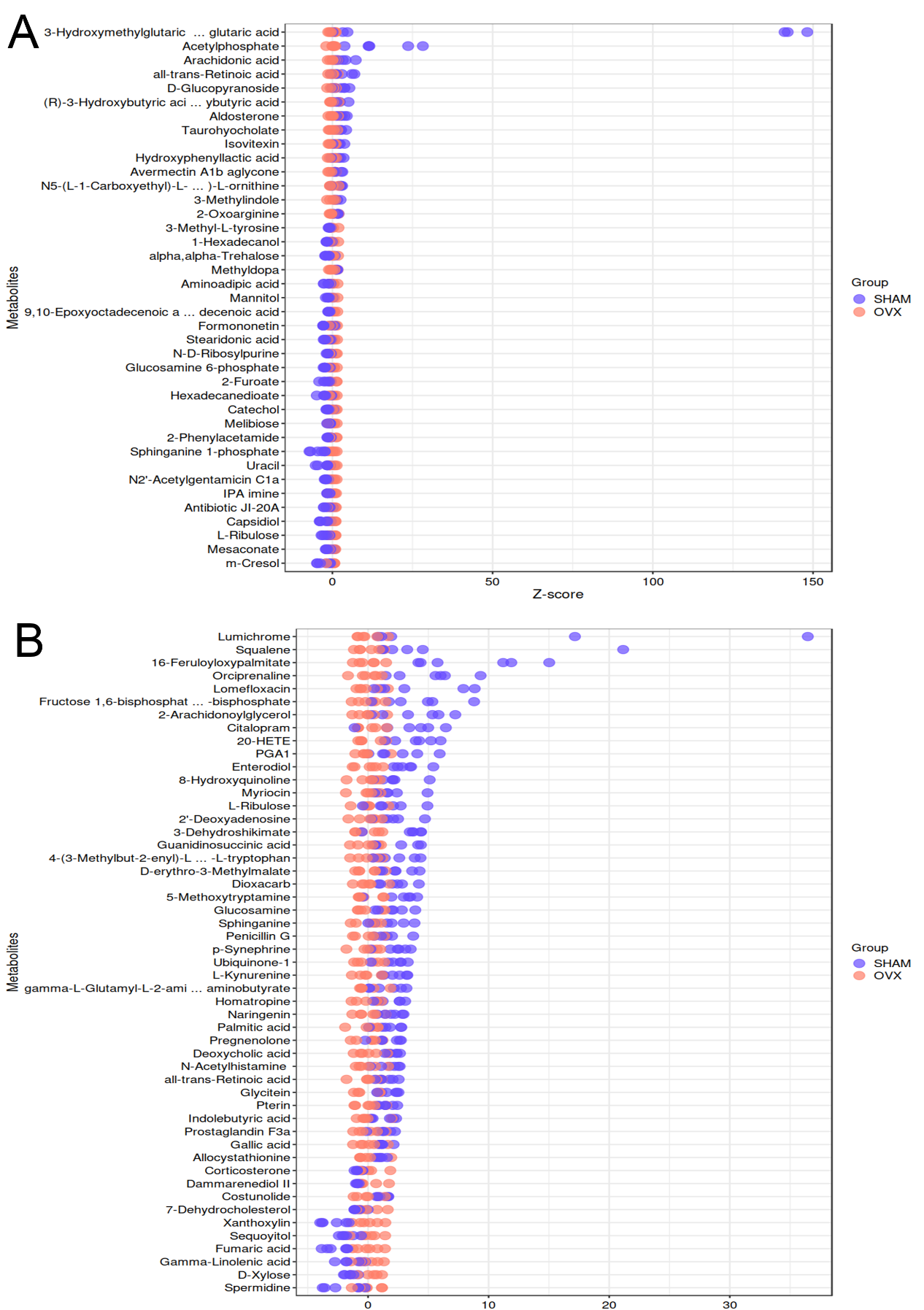


Figure S2 Metabolite differences between the SHAM and OVX groups. (A) Serum. (B) Fecal.

| TableS1 | SHAM vs OVX | | | | | | DMY_H vs OVX | | | | | |
| --- | --- | --- | --- | --- | --- | --- | --- | --- | --- | --- | --- | --- |
| name | FC | log2FC | P.value | -log10(P.value) | FDR | VIP | FC | log2FC | P.value | -log10(P.value) | FDR | VIP |
| Catechol | 0.62 | -0.69 | 0.0318507 | 1.5 | 0.4808743 | 1.6504199 | 0.66 | -0.61 | 0.0387276 | 1.41 | 0.4551602 | 1.6310028 |
| Mannitol | 0.64 | -0.65 | 0.02703 | 1.57 | 0.4808743 | 1.6960322 | 0.55 | -0.85 | 0.001939 | 2.71 | 0.1939297 | 2.184561 |
| N5-(L-1-Carboxyethyl)-L-ornithine | 1.75 | 0.81 | 0.0159625 | 1.8 | 0.4808743 | 1.8063037 | 1.63 | 0.7 | 0.0239637 | 1.62 | 0.4210484 | 1.7689147 |
| Antibiotic JI-20A | 0.58 | -0.78 | 0.0359317 | 1.44 | 0.4808743 | 1.6323412 | 0.45 | -1.15 | 0.0029086 | 2.54 | 0.2327165 | 2.1059473 |
| N2'-Acetylgentamicin C1a | 0.35 | -1.52 | 0.0003896 | 3.41 | 0.1558462 | 2.2929058 | 0.61 | -0.72 | 0.0463348 | 1.33 | 0.4586131 | 1.5289008 |
| 2-Furoate | 0.67 | -0.58 | 0.0229714 | 1.64 | 0.5023102 | 1.8597104 | 0.64 | -0.64 | 0.0371971 | 1.43 | 0.6480417 | 1.95876 |
| IPA imine | 0.69 | -0.53 | 0.0102513 | 1.99 | 0.437307 | 1.8966342 | 0.73 | -0.45 | 0.0277065 | 1.56 | 0.6480417 | 1.8235949 |
| Melibiose | 0.74 | -0.44 | 0.0411252 | 1.39 | 0.5309174 | 1.7443707 | 0.67 | -0.57 | 0.0098089 | 2.01 | 0.6215491 | 2.1461089 |
| Lomefloxacin | 1.64 | 0.71 | 0.0197859 | 1.7 | 0.6502914 | 2.0238239 | 1.61 | 0.69 | 0.0145113 | 1.84 | 0.49176 | 1.9578032 |
| 4-(3-Methylbut-2-enyl)-L-tryptophan | 1.92 | 0.94 | 0.0185908 | 1.73 | 0.6127159 | 1.9640003 | 1.95 | 0.97 | 0.0133922 | 1.87 | 0.3683627 | 1.8956478 |
| 20-HETE | 4.92 | 2.3 | 0.0015601 | 2.81 | 0.3528524 | 2.389462 | 2.29 | 1.19 | 0.0307462 | 1.51 | 0.4502584 | 1.8321542 |
| PGA1 | 2.27 | 1.18 | 0.0131627 | 1.88 | 0.5692102 | 2.006553 | 1.76 | 0.82 | 0.0114791 | 1.94 | 0.3601503 | 1.9149822 |
| Squalene | 2.61 | 1.38 | 0.0194562 | 1.71 | 0.6193166 | 1.891123 | 1.97 | 0.98 | 0.0360483 | 1.44 | 0.4697744 | 1.7021877 |
